# Supplementary material for: The interactome of intact mitochondria by cross-linking mass spectrometry provides evidence for coexisting respiratory supercomplexes
Source: Mol Cell Proteomics. 2017 Dec 8;17(2):216–32. doi: 10.1074/mcp.RA117.000470 (PMC5795388; doi:10.1074/mcp.RA117.000470)
Supplement: Supplemental Data [file supp_RA117.000470_133922_0_supp_23978_fzffwf.zip › spectra_annotation/mito_spectra_annotation/14-1-8-1-1-1.pdf]

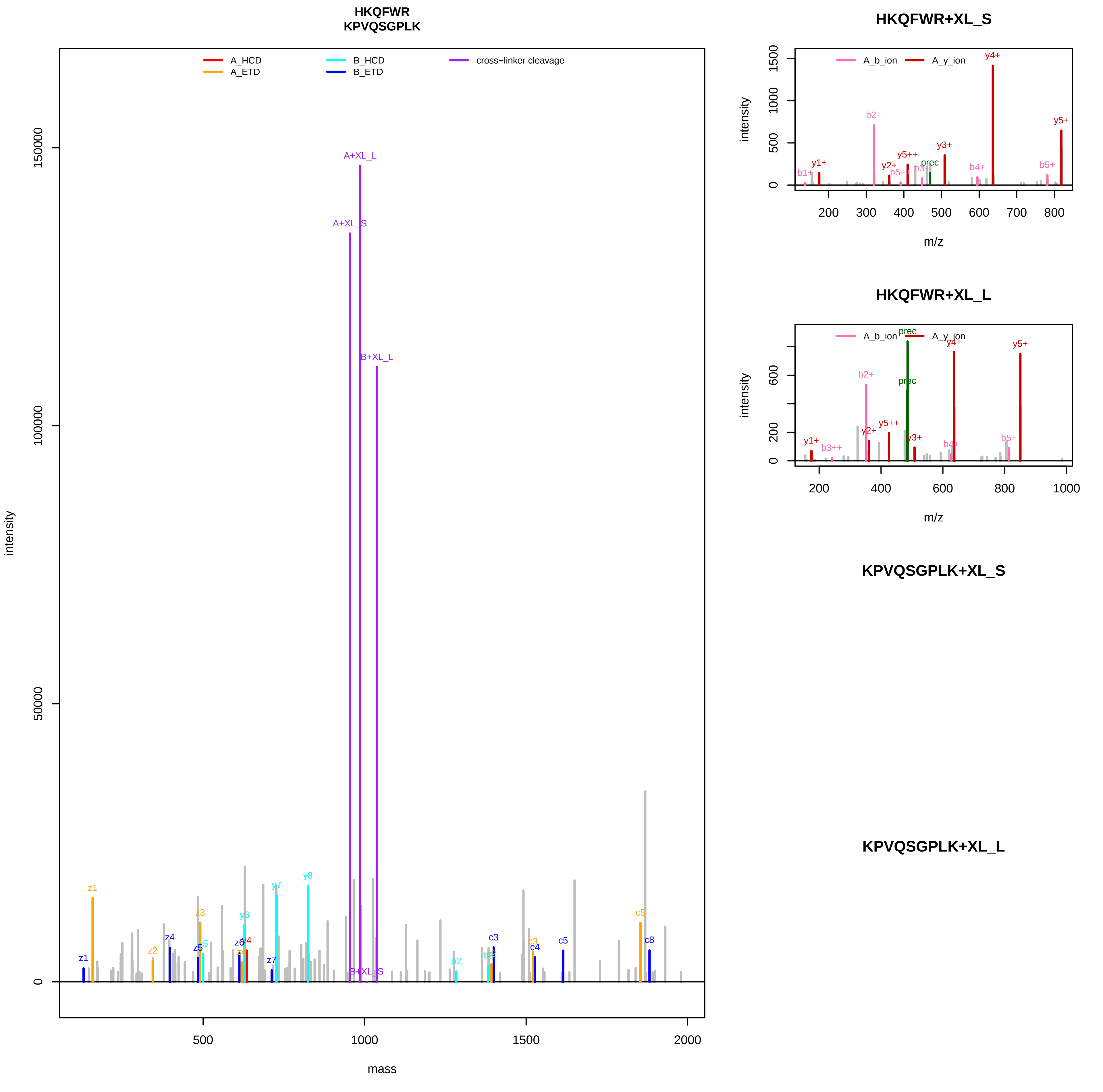
HKQFWR
KPVQSGPLK
A_HCD
A_ETD
B_HCD
B_ETD
cross−linker cleavage
A+XL_L
A+XL_S
B+XL_L
B+XL_S
intensity
mass
0
50000
100000
150000
500
1000
1500
2000
z1 z2 z3 z4 z5 z6 z7 y4 y5 y6 y7 y8 b2 c3 c4 c5 c8
HKQFWR+XL_S
HKQFWR+XL_L
A_b_ion
A_y_ion
prec
b1+ b2+ b3+ b4+ b5+ b3++ b5++
y1+ y2+ y3+ y4+ y5+ y5++
intensity
m/z
0
500
1000
1500
200
300
400
500
600
700
800
0
200
600
1000
KPVQSGPLK+XL_S
KPVQSGPLK+XL_L
